# Clinical manifestations of COVID-19 in patients with asthma, hypertension, and diabetes mellitus

**DOI:** 10.25122/jml-2021-0364

**Published:** 2022-12

**Authors:** Retno Budiarti, Ediono Ediono, Mohammad Kalaznykov, Yoshio Yamaoka, Muhammad Miftahussurur

**Affiliations:** 1 Department of Microbiology, Faculty of Medicine, Universitas Hang Tuah, Surabaya, Indonesia; 2 Faculty of Medicine, Universitas Hang Tuah, Surabaya, Indonesia; 3 Department of Environmental and Preventive Medicine, Oita University, Faculty of Medicine, Yufu, Japan; 4 Gastroentero-Hepatology Division, Department of Internal Medicine, Faculty of Medicine-Dr. Soetomo Teaching Hospital, Universitas Airlangga, Surabaya, Indonesia; 5 Helicobacter pylori and Microbiota Study Group, Institute of Tropical Disease, Universitas Airlangga, Surabaya, Indonesia

**Keywords:** COVID-19, comorbidity, asthma, hypertension, DM

## Abstract

The clinical symptoms of COVID-19 infection differ from one patient to another, requiring different management. This study intended to characterize the clinical manifestation of COVID-19 in patients with asthma, hypertension, and diabetes mellitus (DM). We analyzed data from 540 medical records of patients with comorbidities like asthma, hypertension, and DM diagnosed with COVID-19, looking at vital signs upon admission, chest X-ray, and laboratory results. Most patients were male (57.0%). The most prevalent comorbidity was hypertension (46.3%) and DM (46.3%), followed by asthma (7.4%). 273 patients had improved condition (50.6%). Patients with hypertension had the most extended length of stay compared to other comorbidities groups (13.0±8.5 days). There were significant differences in the oxygen saturation and respiration rate between the three groups (p=0.002, p<0.0001, respectively). The mean lymphocyte (p=0.028) and hematocrit count (p=0.001) were significantly different between the three comorbid groups, with the hypertension group having the highest mean lymphocyte (15.1±8.9) and hematocrit (38.7±6.9) count. COVID-19 had a significant impact on patients with asthma, hypertension, and diabetes comorbidities concerning the final condition, length of stay, oxygen saturation, and respiratory rate, and also on the hematology level, mainly lymphocyte and hematocrit. Treatment, age, and infection might be determinant factors for different outcomes in each type of comorbidity.

## INTRODUCTION

In December 2019, unknown pneumonia cases were reported in Wuhan, China. The National Health Commission (NHC) announced that the cause was SARS-CoV-2 [1, 2]. Following this, the World Health Organization (WHO) labeled SARS-CoV-2 as COVID-19 disease with the potential to become a severe disease. The WHO determined that SARS-CoV-2 was a pandemic, referring to more than 118,000 cases of coronavirus disease in more than 110 countries and regions around the world [3]. All countries had to respond quickly to the spread of COVID-19 to protect vulnerable groups and avoid fatalities [4].

SARS-CoV-2 is a positive sense single-stranded RNA virus belonging to the genus *Betacoronavirus* in the family *Coronaviridae*. It is genetically identical to Severe Acute Respiratory Syndrome (SARS) in 79% of cases and Middle East Respiratory Syndrome (MERS) in 50% of cases, and it originated in *Malayan pangolins* (*Manis Javanica*) and *bats* (*Rhinolophulus affinis*) [1, 5, 6]. However, COVID-19 disease has different clinical manifestations. It was reported that about 51% of patients had comorbidities, including diabetes mellitus (DM) (10–20%), hypertension (10–15%), and cerebrovascular and cardiovascular disease (7–40%) [7–9].

Although SARS-CoV-2 shares many of the same virological characteristics as MERS and SARS, SARS-CoV-2 is highly contagious. COVID-19 symptoms include mild upper respiratory tract infections, severe pneumonia, and death due to acute respiratory failure. Numerous host factors, such as advanced age, smoking, and comorbidities, have been implicated in the prognosis of COVID-19 [10, 11]. SARS-CoV-2 infects people of all ages, but those over 60 are at a higher risk of infection, as are those with chronic respiratory disease, DM, or cardiovascular disease [12].

It can be observed from the emerging cases that comorbidity increases the chance of infection [13]. Individuals of all ages with serious underlying illnesses, as well as the elderly, particularly those in long-term care facilities, are more likely to contract COVID-19 [14]. Hypertension, DM, chronic obstructive pulmonary disease (COPD), cardiovascular and cerebrovascular disease were identified as significant risk factors for COVID-19 in a meta-analysis. Awareness of these risk factors may help identify COVID-19 patients at increased risk, allowing for a more targeted and specific approach to prevent death [15]. In addition, individuals with uncontrolled medical conditions such as hypertension, DM, liver, lung, and kidney disease, transplant recipients, smokers, cancer patients undergoing chemotherapy, and chronic steroid users are at an increased risk of COVID-19 infection [14, 16].

COVID-19 infection induces an inflammatory response in patients. The inflammatory response plays a critical role in COVID-19, and inflammatory cytokine storm increases severity [17]. Some hematological parameters, including white blood cell (WBC), lymphopenia, C-reactive protein (CRP), and some biochemical parameters, such as lactate dehydrogenase (LDH), creatine kinase (CK), and troponin, were reported to be associated with COVID-19 severity [18]. This study investigated the effect of COVID-19 infection on the clinical condition of patients with hypertension, DM, and asthma comorbidities.

## MATERIAL AND METHODS

This was a cross-sectional analytic observational study. The population included patients diagnosed with COVID-19 at Dr. Ramelan Hospital, Surabaya, Indonesia, for 5 months (March-August 2021) with comorbid asthma, hypertension, and DM. This study used a total sampling of all patients diagnosed by clinicians with COVID-19 based on clinical symptoms and laboratory results in the form of a positive antigen swab within five months (March-August 2021). Inclusion criteria in this study included patients diagnosed with suspected COVID-19, SARS CoV-2 by a pulmonary or internal medicine clinician at Dr. Ramelan Hospital, Surabaya, with comorbid DM, hypertension, and asthma. The exclusion criteria were patients with incomplete medical records.

### Research procedure

Patients with complaints such as shortness of breath, cough, runny nose, and fever with or without anosmia were examined by clinicians at Dr. Ramelan Hospital Surabaya, who performed a physical examination and offered the necessary support. Patients were diagnosed with suspected COVID-19, then an antigen swab test was carried out by taking an oropharyngeal swab sample. If the result was positive, they were included in the study sample. Following anamnesis, patients with asthma, hypertension, and DM were included in the group with comorbidities. Patients were followed during hospitalization in the COVID-19 room, and the progression of symptoms was observed through several parameters in the next 7 to 10 days. Data were tabulated and analyzed among the three comorbid groups.

### Statistical analysis

Statistical analysis was performed using IBM SPSS^®^ Statistics Version 22 (IBM Corp., USA). Demographic and clinical characteristics were presented descriptively using frequency and percent for the type of data category (nominal and ordinal). Continuous data (intervals and ratios) are shown as mean±standard deviation (SD). Tukey's test, Chi-Square test, Kruskal Wallis test, and ANOVA were used to identify the statistical differences in clinical manifestations between comorbidities. P-value<0.05 was considered statistically significant.

## RESULTS

The total study population included 540 patients, out of which 40 (7.4%) had asthma, 250 patients (46.3%) had hypertension, and 250 patients (46.3%) had DM. Table 1 describes the patient characteristics.

**Table 1:** Patients' characteristics.

Variable	Comorbidities (n=540)			P
	Asthma (n=40)	Hypertension (n=250)	DM (n=250)	
**Age (mean±SD, years)**	46.3±17.4	51±14.3	56±11.1	<0.0001 ^a^ *
**Sex (n, %)**				
Male	24 (4.4)	147 (27.2)	137 (25.4)	0.615 ^b^
Female	16 (6.4)	103 (19.1)	113 (20.9)	-
**Final condition (n, %)**				
Health	9 (1.7)	39 (7.2)	21 (3.9)	0.004 ^b^ *
Ill	0 (0)	1 (0.2)	3 (0.6)	-
Improve	19 (7)	137 (50.2)	117 (42.9)	
Death	12 (6.2)	73 (37.6)	109 (56.2)	
Length of stay (mean±SD, day)	10.1±7.7	13.0±8.5	10.1±6.6	0.001 ^c^ *
**Vital signs (mean±SD)**				
SBP (mmHg)	133.6±18.4	138.1±27.0	141.3±25.8	0.076 ^c^
DBP (mmHg)	83.1±14.4	83.6±17.9	83.8±15.1	0.907 ^c^
Temperature (°C)	36.8±0.8	36.9±0.8	36.9±0.9	0.719 ^c^
Saturation (%)	95.8±5.0	96.0±7.4	94.7±7.6	0.002 ^c^ *
Respiration rate (breaths/minute)	25.1±6.7	23.9±5.2	22.7±4.9	<0.0001 ^c^ *
**Laboratory results (mean±SD)**				
Leukocyte	13.1±9.3	9.9±5.5	11.1±6.5	0.449 ^c^
D-dimer	3189.4±5821.6	2457.7±4474.0	2257.9±3705.9	0.912 ^c^
Hemoglobin (mg/dl)	12.5±2.7	12.8±2.3	12.6±2.5	0.793 ^d^
Lymphocyte	10.8±5.1	15.1±8.9	12.3±8.2	0.028 ^c^ *
Hematocrit	37.7±7.5	38.7±6.9	37.8±7.3	0.001 ^b^ *

a – Tukey Test; ^b^ – Chi-Square Test; ^c^ – Kruskal Wallis Test; ^d^ – ANOVA; SBP – systolic blood pressure; DBP – diastolic blood pressure.

The mean age of patients was 56±11.1 years. There were differences in age between asthma, hypertension, and DM groups (p<0.001). Most patients were males. There were no differences in sex between each comorbidity (p=0.615). Most patients improved (50.6%) and showed differences between the three comorbidities (p=0.004). Patients with hypertension had the most extended length of stay compared to other comorbidities groups (13.0±8.5 days). The statistical test showed differences between groups (p=0.001). Further testing was carried out post hoc (Mann Whitney). There was a difference between the length of stay between COVID-19 patients with asthma and hypertension (p=0.04) and DM and hypertension (p<0.0001).

We also observed vital signs in COVID-19 patients with all three comorbidities. Oxygen saturation and respiration rates were significantly different in the three groups (p=0.002, p<0.0001, respectively). However, there were no significant differences in the value of systole (p=0.076), diastole (p=0.907), and temperature (p=0.719). The laboratory results were recorded when the patient first entered the hospital and was clinically diagnosed with COVID-19. In this study, the chest X-ray picture of comorbid asthma did not significantly differ from other comorbidities, and the chest X-ray picture of pneumonia was not specific for each comorbidity. There were significant differences in the mean lymphocyte (p=0.028) and hematocrit count (p=0.001) among the three comorbid groups, with the hypertension group having the highest mean lymphocyte (15.1±8.9) and hematocrit (38.7±6.9) count compared to the other groups.

## DISCUSSION

We identified the clinical features of COVID-19 patients with asthma, hypertension, and diabetes mellitus (DM) comorbidities. We found that oxygen saturation, respiration rate, lymphocyte count, and hematocrit were significantly different among the three groups. Compared to other underlying diseases, common comorbid diseases such as COPD, hypertension, diabetes, and cardio-cerebrovascular disease were more significant risk factors in COVID-19 patients [14]. Patients who have at least one comorbidity or more have poor clinical outcomes [7]. One of the most frequently observed laboratory abnormalities is a decreased lymphocyte count, which increases the risk of acute respiratory distress syndrome (ARDS) and, thus, the severity of the disease in COVID-19 patients [19–21].

The final condition in each comorbidity might be influenced by treatment and the host condition. The majority of patients who were getting better had hypertension. Previous studies revealed no significant relationship between hypertension and blood pressure with the severity of COVID-19 [22]. The increase in the patient's blood pressure is not always accompanied by the severity of the disease. This might be due to the anti-hypertensive treatment that plays a role in the renin-angiotensin system (RAS) [23]. Some previous observational studies identified that RAS inhibition treatment might even reduce the risk of severe COVID-19 or mortality [24]. The worse outcome mainly occurred in diabetic patients. Diabetes mellitus (DM) and bad glucose control are significant factors for higher risk of poor COVID-19 outcomes [25]. A meta-analysis study suggested DM was associated with a significantly increased mortality risk compared to that observed in the nondiabetic population [26].

Diabetic patients have elevated plasma glucose which influences SARS-CoV-2 replication through a mechanism of mitochondrial reactive oxygen species production and activation of hypoxia-inducible factor 1α [27]. Therefore, the viral proliferation might also have been encouraged by hyperglycemia. A previous study revealed that hyperglycemia or a history of type 1 diabetes mellitus (T1DM) and type 2 diabetes mellitus (T2DM) were independent predictors of morbidity and mortality in patients with SARS [28].

Hypertensive patients have the most extended average hospitalization compared to diabetic and asthmatic patients. COVID-19 patients with hypertension or diabetes had 1.82 times increased likelihood of prolonged COVID-19-related length of stay compared to those with no comorbidities (95% CI=1.42–2.33, p=0.00) [29]. Another study also found that hospitalization duration was significantly associated with hypertension in COVID-19 patients [30]. An observational study compared COVID-19 patients with and without hypertension. Hypertensive patients had greater disease severity and adverse progression than non-hypertensive patients. However, the multivariate analysis revealed that hypertension was not the independent factor for severity and mortality. Patients with hypertension need additional treatment to prevent adverse outcomes [31]. Therefore, it can be reasoned that hypertensive patients have a longer duration of hospitalization without high mortality cases.

Vital signs examination showed that COVID-19 patients with diabetes have the lowest average oxygen saturation and respiratory rates. Interestingly, asthma patients had the highest average respiratory rate. A study on 72 COVID-19 patients with asthma revealed that asthmatic comorbidity was neither associated with increased severity nor worse outcomes [32].

The result might be influenced by demographical factors such as the age of patients. The highest average age was found in patients with diabetes comorbidity. COVID-19 patients aged 50 showed a statistically significant increased risk of severity (3.36 times) compared to those below 50 (RR=3.36; 95% CI=1.79–6.30, p=0.0002) [33].

The laboratory examination results revealed two significant differences in lymphocyte and hematocrit levels. In the human body, lymphocytes are the most important immune cells that regulate cellular immunity [34]. Many studies have shown that a reduction in the total count of lymphocytes indicates that the coronavirus has consumed many immune cells and is common in critically ill patients [35]. The current study found that lymphocyte and hematocrit levels were low in all comorbidities compared with the normal range. Lower lymphocytes, RBCs, hemoglobin, and hematocrit levels were found in the severe group of COVID-19 patients at the end of treatment [36].

Although this study did not find significant differences in leukocyte and d-dimer levels, their values increased. These could explain the more aggravated inflammation storm and severe multiorgan injuries in COVID-19 patients [37]. Additionally, previous studies reported that elevated d-dimer and leukocyte levels are associated with adverse outcomes in COVID-19 patients [38].

One of the limitations of this study is that it only documented clinical data from medical records and was based on daily observation. The focus was not on the treatment protocols that might influence the clinical outcomes of patients. The pathological alteration of patients was also not recorded in the questionnaire list. In addition, parameters like vitamin D levels and ferritin associated with COVID-19 progression were not identified in this study. Future studies should cover all parameters that could have a significant role in COVID-19 clinical manifestation, using a complete protocol and the right methods.

## CONCLUSION

Patients with COVID-19 and asthma, hypertension, and diabetes comorbidities had significant differences in the final condition, length of stay, oxygen saturation and respiratory rate, and hematology levels, mainly lymphocyte and hematocrit count. The treatment, age, and infection condition might be a determinant factor for the emergence of different outcomes in each type of comorbidity.
